# Inter‐ and intrafractional dose uncertainty in hypofractionated Gamma Knife radiosurgery

**DOI:** 10.1120/jacmp.v17i2.5851

**Published:** 2016-03-08

**Authors:** Taeho Kim, Jason Sheehan, David Schlesinger

**Affiliations:** ^1^ Department of Radiation Oncology University of Virginia Health System Charlottesville VA; ^2^ Department of Neurological Surgery University of Virginia Health System Charlottesville VA; ^3^ Department of Radiation Oncology Virginia Commonwealth University Richmond VA USA

**Keywords:** dose uncertainty analysis, hypofractionated radiosurgery, Gamma Knife radiosurgery, Extend system

## Abstract

The purpose of this study is to evaluate inter‐ and intrafractional dose variations resulting from head position deviations for patients treated with the Extend relocatable frame system utilized in hypofractionated Gamma Knife radiosurgery (GKRS). While previous reports characterized the residual setup and intrafraction uncertainties of the system, the dosimetric consequences have not been investigated. A digital gauge was used to measure the head position of 16 consecutive Extend patients (62 fractions) at the time of simulation, before each fraction, and immediately following each fraction. Vector interfraction (difference between simulation and prefraction positions) and intrafraction (difference between postfraction and prefraction positions) shifts in patient position were calculated. Planned dose distributions were shifted by the offset to determine the time‐of‐treatment dose. Variations in mean and maximum target and organ at risk (OAR) doses as a function of positional shift were evaluated. The mean vector interfraction shift was 0.64 mm (Standard Deviation (SD): 0.25 mm, maximum: 1.17 mm). The mean intrafraction shift was 0.39 mm (SD: 0.25 mm, maximum: 1.44 mm). The mean variation in mean target dose was 0.66% (SD: 1.15%, maximum: 5.77%) for interfraction shifts and 0.26% (SD: 0.34%, maximum: 1.85%) for intrafraction shifts. The mean variation in maximum dose to OARs was 7.15% (SD: 5.73%, maximum: 30.59%) for interfraction shifts and 4.07% (SD: 4.22%, maximum: 17.04%) for intrafraction shifts. Linear fitting of the mean variation in maximum dose to OARs as a function of position yielded dose deviations of 10.58%/mm for interfractional shifts and 7.69%/mm for intrafractional shifts. Positional uncertainties when performing hypofractionated Gamma Knife radiosurgery with the Extend system are small and comparable to frame‐based uncertainties (<1 mm). However, the steep dose gradient characteristics of GKRS mean that the dosimetric consequences of positional uncertainties should be considered as part of treatment planning. These dose uncertainties should be evaluated in the context of tumor response and OAR tolerance for hypofractionated treatment scenarios where any increase in dose may be tempered by the increased protection hypofractionation provides to normal tissue.

PACS number(s): 87.52.‐g

## I. INTRODUCTION

Gamma Knife radiosurgery (GKRS) delivers high doses of radiation to a specified target while sparing healthy surrounding tissues.^(1)^ Traditionally, GKRS is a single‐session procedure utilizing a rigid frame‐based technique for both defining a stereotactic coordinate system for targeting, as well as for immobilizing, the patient's head. The frame is rigidly fixed to the patient via four pins which attach the frame to the outer table of the patient's skull.^(2)^ While this is a key feature that has historically made single‐session intracranial radiosurgery possible, repeatedly reapplying an invasive frame for hypofractionated procedures is not practical.

**Figure 1:**
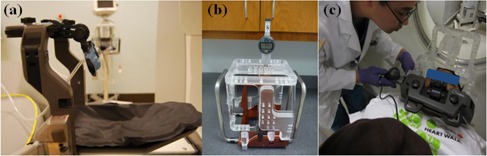
Extend frame system (a), measurement gauges (b), and template (c); patient undergoing position measurement at time of simulation imaging. The mean vector setup shift (Eq. (1)) was 0.64 mm (SD: 0.25). The mean intrafractional shift was 0.39 mm (SD: 0.25).

Recently, the Extend system for the Gamma Knife Perfexion (Elekta Instruments, AB, Stockholm, Sweden) has been developed for hypofractionated GKRS procedures.^3^, ^4^, ^5^ The Extend system consists with a vacuum‐assisted dental fixation device attached on a carbon‐fiber frame for noninvasive immobilization in Fig. 1(a) and a linear measurement system for patient repositioning in Fig. 1(b).

In the clinical setting, recently, Schlesinger et al.^(6)^ have quantified the uncertainty for patient setup and patient immobilization when the Extend system is used in the hypofractionated GKRS procedures. In the report, they evaluated the performance of the Extend system in terms of interfractional variation between each fraction and intrafractional variation during each fraction for 10 consecutive patients (total 36 fractional treatments). In the study, reference measurements of the patient's position within the Extend frame were taken at the time of CT simulation. Positional measurements were then taken before delivery of each fraction (prefraction) and following delivery of each fraction (postfraction). The study reported a mean vector setup difference (Eq. (1))^(6)^ of 0.64 mm and a mean intrafractional positional difference of 0.47 mm.

(1) $$\Delta_{vector}=\sqrt{{\left(\Delta_{anterior}\right)}^{2}+{\left(\Delta_{\text{superior}}\right)}^{2}+{\left((\Delta_{left}-\Delta_{right})/2\right)}^{2}}$$

where $\Delta_{anterior}$, $\Delta_{\text{superior}}$, $\Delta_{left}$, and $\Delta_{right}$ are the mean values for each plate of the RCT.

While the previous report characterized the residual setup and intrafraction uncertainties of the system, the dosimetric consequences of these uncertainties have not been fully investigated. In this study, we explored the relationship between interfraction and intrafraction head position uncertainty, and dosimetric uncertainty using clinical data from hypofractionated Gamma Knife radiosurgery Extend system treatments.

## II. MATERIALS AND METHODS

### A. Setup uncertainty and intrafraction uncertainty

This study was an institutional review board‐approved, retrospective examination of prospectively acquired data from 16 consecutive Gamma Knife Extend patients with a total of 64 treatment fractions. Table 1 details the tumor type, tumor location, tumor volume, prescription dose, closest organ at risk (OAR), and minimum distance to the closest OAR. Using the digital linear measurement gauges and measurement template included with the Extend system in Fig. 1, the head positions of 16 patients who underwent hypofractionated GKRS were recorded. The digital linear measurement probes (C150XB Digimatic Indicator, Mitutoyo America Corp., Aurora, IL) and the repositioning check tool (RCT) as a measurement template were used to measure the head positions of the patients. The RCT mounted on the Extend frame over the patient's head has several measurement locations on its superior, anterior, left, and right plates. Using the measurement probes, measurements of the head position were taken with 0.01 mm resolution and 0.006 mm accuracy (manufacturer specifications).^6^, ^7^ Measurements were taken at the time of simulation imaging, immediately before delivery of each fraction (prefraction), and immediately following each fraction (postfraction), Fig. 1(c). Residual setup (interfraction) shift was defined as the remaining difference after patient setup between the prefraction head position and the head position at time of simulation imaging. Intrafraction shift was defined as the difference between the postfraction head position and the prefraction head position.^(8)^

**Table 1:** Demographics: the tumor type, tumor location, tumor volume, prescription dose, closest organ at risk (OAR), and minimum distance to the closest OAR (PIL)

*Patient ID*	*Tumor Type*	*Tumor Location*	*Prescription Dose (Gy)*	*# Fraction*	*Prescription Isodose Volume (cc)*	*Tumor Volume (GTV) (cc)*	*OAR*	*PIL (mm)*
1	Meningioma	anterior clinoid	20	4	2.99	2.45	optic pathway	0.0
2	Meningioma	cavernous sinus	18	3	15.71	11.44	optic pathway	0.0
3	Meningioma	cavernous sinus	18	3	9.21	7.16	optic pathway	3.2
4	Meningioma	sphenoid wing	20	4	5.78	5.04	optic pathway	1.6
5	5th nerve schwannoma	petroclival	20	4	9.93	8.42	brainstem	0.0
6	Meningioma	cavernous sinus	20	4	5.72	4.11	optic pathway	0.3
7	Meningioma	cavernous sinus	18	4	11.98	11.22	optic pathway	2.5
8	Meningioma	falcine	18	3	22.69	21.3	none	‐
9	Meningioma	sphenoid wing	20	4	1.35	1.79	optic pathway	0.0
10	Meningioma	tuberculum sellae	18	3	1.3	0.94	optic pathway	2.0
11	Meningioma	clinoid	20	4	0.31	0.2	optic pathway	1.0
12	Meningioma	sphenoid wing	20	5	19.62	16.45	optic pathway	0.7
13	Meningioma	cavernous sinus	20	5	1.1	1.1	optic pathway	0.3
14	Meningioma	clival	25	4	8.95	7.45	brainstem	0.0
15	Pituitary adenoma	sella / cavernous sinus	25	5	6.41	5.78	optic pathway	2.7
16	Meningioma	cavernous sinus	15	5	7.3	6.32	optic pathway	1.0

### B. Dosimetric variation due to the inter‐ and intrafraction position changes

**Figure 2:**
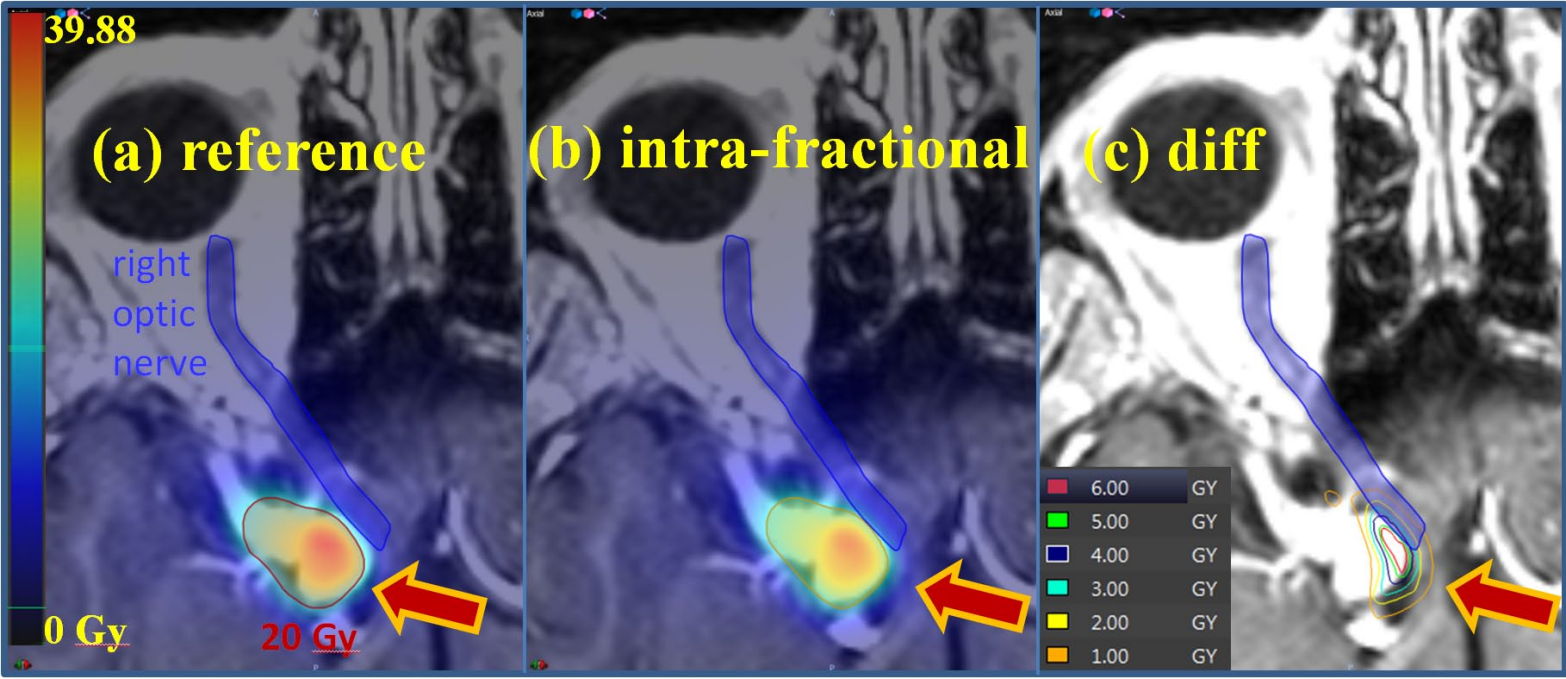
Reference dose distribution (a), dose distribution after intrafraction shift (b), and (c) dose difference between (a) and (b). The red contour is the 20 Gy isodose line on the target (arrow) and the blue contour is the right optic nerve in (a) and (b). For this example, the prescription dose is 20 Gy and the maximum dose is 39.88 Gy within the target. The vector setup difference is 0.83 mm, visible as a shift in the location of the higher isodose regions towards the patient's midline. In (c), isodose lines demonstrate higher than planned dose to the right optic nerve due to the positional variation.

The dosimetric consequences of the inter‐ and intrafractional uncertainty were investigated in terms of the geometric relationship between the dose distribution and nearby radiosensitive normal tissue structures (most frequently the optic nerves). The dose distribution and any contoured structures from the reference treatment plan (which is based on patient position at the time of simulation imaging) were exported from the Gamma Knife treatment planning system in DICOM‐RT format.^(9)^ For each treatment fraction delivered with a particular treatment plan, the planned dose distributions were shifted by the clinically measured offset between the prefraction and reference head positions in order to determine the variation in dose due to interfraction uncertainty. Likewise, for each treatment plan the dose distributions were shifted by the clinically measured offset between the post‐treatment and pretreatment head positions to determine the variation in dose due to intrafraction uncertainty. In the study, adjusted dose distributions for each interfraction and intrafraction shift were created using a commercial registration tool (VelocityAI 3.0.1; Varian Medical Systems, Palo Alto, CA). Each shift was applied to the reference dose distribution relative to the planning MRI during coregistration in Fig. 2.

### C. Correlation of the residual motion to dosimetric variation

Dose metrics including minimum dose, maximum dose, and mean dose were computed for the target and any nearby contoured organs at risk (OARs). Percentage changes in these dose metrics were determined as a linear function of the magnitude of the patient position variation.

## III. RESULTS

### A. Setup and intrafraction uncertainties and the dosimetric consequences

The mean vector interfraction shift was 0.64 mm (standard deviation (SD): 0.25 mm, maximum: 1.17 mm). The mean intrafraction shift was 0.39 mm (SD: 0.25 mm, maximum: 1.44 mm). Across all fractions, the mean variation in mean target dose was 0.66% (SD: 1.15%, maximum: 5.77%) due to interfraction shifts and 0.26% (SD: 0.34%, maximum: 1.85%) due to intrafraction shifts, shown in Table 2. The mean variation in mean dose to OARs was 3.98% (SD: 3.16%, maximum: 13.96%) due to interfraction shifts and 2.67% (SD: 2.09%, maximum: 9.02%) due to intrafraction shifts. The mean variation in maximum dose to OARs was 7.15% (SD: 5.73%, maximum: 30.59%) due to interfraction shifts and 4.07% (SD: 4.22%, maximum: 17.04%) due to intrafraction shifts.

**Table 2:** Dosimetric comparison to tumor and OAR: [Average (STD)] cGy. Percentage differences from the reference (%) are presented

*Parameter*	*Reference Dose (Gy)*	*Dose After Interfractional Shift (Gy)*	*Percentage Difference Inter‐(%)*	*Dose After Intrafractional Shift (Gy)*	*Percentage Difference Intra‐(%)*
*Tumor*					
Mean	26.44(3.26)	26.29(3.37)	0.66(1.15)	26.40 (3.27)	0.26 (0.34)
*OAR*					
Mean	6.11(2.69)	6.04(2.71)	3.98(3.16)	6.14(2.78)	2.67 (2.09)
Max	17.53 (4.78)	17.26 (4.53)	7.15(5.73)	17.62(5.04)	4.07 (4.22)

### B. Correlation of the residual motion to dosimetric variation

**Figure 3:**
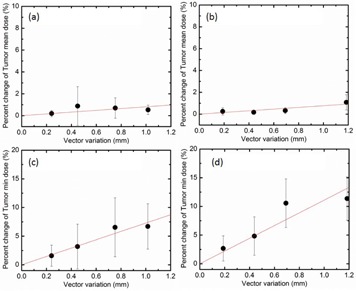
Correlation of the residual motion with 0.3 mm bin size to dosimetric variation with interfractional uncertainty and with intrafractional uncertainty: (a) the mean variation in mean target dose with interfractional uncertainty; (b) the mean variation in mean target dose with intrafractional uncertainty; (c) the mean variation in minimum target dose with interfractional uncertainty; (d) the mean variation in minimum target dose with intrafractional uncertainty. The red solid line is the linear fit to the measured data. $R^{2}$: (a) 0.42, (b) 0.79, (c) 0.91, (d) 0.78.

Linear fittings of the mean variation in mean target dose, in mean dose to OARs and in maximum dose to OARs as a function of position were performed. Mean target dose deviations as a function of the position changes are 0.81%/mm for interfractional shifts and 0.77%/mm for intrafractional shifts in Figs. 3(a) and (b). Minimum target dose deviations as a function of the position changes are 7.29%/mm for interfractional shifts and 11.09%/mm for intrafractional shifts in Figs. 3(c) and (d). The red solid line is the linear fit to the measured data.

**Figure 4:**
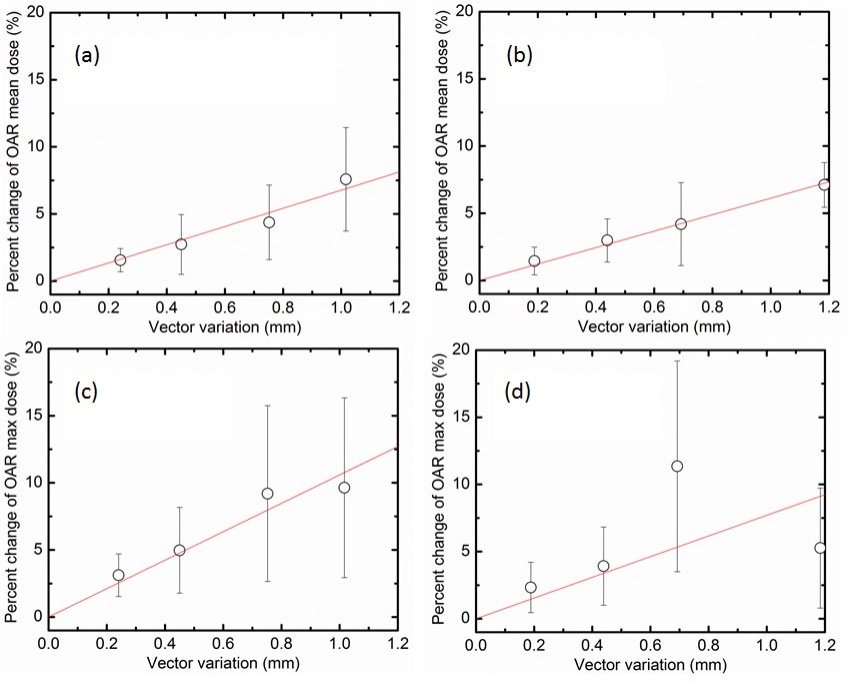
Correlation of the residual motion with 0.3 mm bin size to dosimetric variation with interfractional uncertainty and with intrafractional uncertainty: (a) the mean variation in mean dose to OARs with interfractional uncertainty; (b) the mean variation in mean dose to OARs with intrafractional uncertainty; (c) the mean variation in maximum dose to OARs with interfractional uncertainty; (d) the mean variation in maximum dose to OARs with intrafractional uncertainty. The red solid line is the linear fit to the measured data. $R^{2}$: (a) 0.95, (b) 0.99, (c) 0.90, (d) 0.12.

In Fig. 4, the mean variation in mean dose to OARs was 6.77%/mm for interfractional shifts and 6.11%/mm for intrafractional shifts. The mean variation in maximum dose to OARs near the tumor was 10.58%/mm for interfractional shifts and 7.69%/mm for intrafractional shifts. The difference of the mean variation in maximum dose to OARs due to inter‐ and intrafraction shifts is likely due to differences in the directions of the shifts relative to OAR geometry. The minimum distance between the prescription isodose line (PIL) to nearby OARs was 1.0 mm (SD: 1.1 mm).

## IV. DISCUSSION

In the study, we investigated the relationship between interfraction and intrafraction head position uncertainty, and dosimetric uncertainty using clinical data from hypofractionated Gamma Knife Radiosurgery Extend system treatments.

Over 16 consecutive Extend patients, we found 0.64 mm mean vector inter‐fractional difference and 0.39 mm mean vector intrafractional difference. These results are in good agreement with the previous reports by Schlesinger et al.^(6)^ and Ma et al.,^(10)^ and the additional six patients to Schlesinger's study included in the study did not significantly alter the previously reported results. It should be noted that when setting up a patient, the patient position generally corrected if the vector difference between the setup and reference head positions were larger than 1 mm. In 5 fractions for four patients, a clinical decision was made to proceed with a treatment with residual setup differences of slightly more (maximum=1.17 mm) than 1.0 mm. The interfraction uncertainty is therefore bounded by a 1.0 mm ceiling, and really represents the residual uncertainty that remains after positioning the patient to within a clinically meaningful threshold. It should be noted that the digital measurement gauges and RCT do not directly measure rotations. However, multiple measurement points (typically three points) are measured on each measurement plate and then averaged. Unless a head is spherical, the irregularities of the head shape will implicitly capture some rotational error. In this study, we acquired data on actual patients using clinical procedures and the clinical Extend system and as such, we did not explicitly measure rotation.

The dosimetric consequences these small interfractional and intrafractional shifts cause only insubstantial variations in mean tumor dose (mean=0.66% due to interfractional variations and mean=0.26% due to intrafractional variations). The magnitudes of the interfractional and intrafractional shifts are generally much smaller than the diameter of the tumors treated.^11^, ^12^ GKRS dose distributions are designed to cover substantially all of the tumor volume at the prescription isodose level (with maximum dose points that lie inside the tumor), so the relatively small shifts in dose distribution relative to the tumor will have little statistical effect. It is noted that these mean tumor dose uncertainties are still correlated with the positional uncertainties. For example, in our study, we found larger interfractional uncertainty than intrafractional uncertainty, resulting in larger tumor dose variation by interfractional shifts.

In contrast to the insubstantial mean tumor dose uncertainty, the mean variation in maximum dose to OARs can be larger (mean of 7.15% due to interfractional shifts and 4.07% due to intrafraction shifts). OARs are by definition geometrically close to the tumor. The high conformity of the dose distributions in GKRS and the sharp pose falloff outside of the target means that while small volumes of OAR may receive some significant dose below a tolerance threshold, the dose gradient across the OAR tends to be quite high. Therefore, small positional variations can cause large differences in dose across an OAR. For example, the dose variation for OARs as a function of shift magnitude can rise as the PIL to OAR distance decreases and the dose gradient increases on OARs. In the study, the minimum distance between the PIL to nearby OARs was 1.0 mm (SD: 1.1 mm) so 1 mm positional shift to the OAR can make dosimetric variation on the OAR which may be of dosimetric and clinical consequence. The results show that positional uncertainties with the Extend system are quite small (<1 mm). The dosimetric consequences for the targeted tumor are also quite small. However, dose variations to nearby OARs may be much more appreciable, especially when the PIL to OAR distance is small.

Using the linear model function, we found the correlation of the residual uncertainty and dosimetric variation. For example, percentage changes in dose metrics were determined as a function of the magnitude of the patient position variation. As shown in Fig. 3, the mean variation in mean target dose as a function of shift is insignificant: 0.81 %/mm for interfractional shifts and 0.77%/mm with intrafractional shifts. However, the mean variation in minimum target dose was 7.29%/mm for interfractional shifts and 11.09%/mm for intrafractional shifts, and the mean variation in maximum dose to OARs was 10.58%/mm for interfractional shifts and 7.69%/mm for intrafractional shifts. The difference in target and OAR dose variation due to inter‐ and intrafractional shifts is likely due to differences in the directions of the shifts relative to target and OAR geometry. It should be noted that the specific variations observed are highly dependent on the direction of shifts relative to the direction of the gradient of the dose distribution and the morphology of the OAR. Of note, the relatively small number of data points and relatively large standard deviations in Figs. 3 and 4 allow for a variety of possible data fitting models. We chose the linear model function for its simplicity as well as its capability in dealing with small distances and positional uncertainties (maximum 1.17 mm and 1.44 mm for inter‐and intrafractional shifts, respectively). Due to this, we believed that the linear model function is a reasonable approach. Our assumption is supported by the report of Fenner et al.,^(11)^ who developed an analytical model of Gamma Knife dose profiles by modeling each individual beam as a top‐hat profile and summing beams in a Gamma Knife geometry. Over small distances (0~2 mm) it seems reasonable to model the dose gradient as a linear function.

The accuracy of dose delivery with the Extend system depends critically on the immobilization capabilities of the Extend frame system and the precision with which the vacuum‐detection system of the Extend patient positioning system (PPS) can detect intrafraction changes in patient position. One limitation of the present study is that intrafraction positional shifts are defined as differences in position between the start and end of each fraction. The manufacturer of the Gamma Knife recently announced a new Gamma Knife model (Gamma Knife Icon, Elekta Instruments, AB)^(13)^ that includes on‐board cone‐beam CT imaging, as well as an optical motion tracking system. This new system has the possibility of providing more direct measurement of intrafraction measurement uncertainty.

The ability to compute changes in dose distributions due to positional changes is potentially limited in precision by the resolution of the underlying dose grid. The Gamma Knife planning system uses a variable calculation grid resolution with a fixed number (31×31×31) of sampling points. The grid is scaled to encompass each target in the treatment plan. For this study, completed plans were exported to a commercial registration tool (Velocity) in DICOM‐RT format using a fixed 1.0 mm resolution. While it is possible that the resolution of the calculation grids for planning and registration are sources of uncertainty in the subsequent calculation of the dosimetric effect on the target and OARs, the uncertainty is likely small relative to other sources of uncertainty such as contouring uncertainty.

In this study, a shift in the dose distribution as we have performed ignores the change in the path length for each beam, which could have some effect on the resulting dose distribution that is not captured by purely shifting the dose distribution itself. However, for this project the commercial standard Gamma Knife treatment planning system (GammaPlan) was used to calculate dose. To the best of our knowledge, GammaPlan does not have functionality to allow a positional shift of the isocenters comprising the treatment plan while maintaining the original dwell times for each isocenter. Instead, GammaPlan will recalculate new dwell times for each isocenter and will renormalize dose to the new maximum dose point in the plan. The result would not be directly comparable to the original treatment plan. Furthermore, the positional shifts for each isocenter in the treatment plan are generally small, and each isocenter represents a position that is the intersection of 192 individual cobalt‐60 beams. Any changes in dose distribution due to changes in individual beam path length are likely to be averaged out by opposite changes in path length from beams coming from the opposite directions. The dosimetric uncertainty that would be caused by plan renormalization and changes in dwell times is likely to be greater than any changes caused by small path‐length changes.

Another limitation of the study is the relatively small number of patients and the probability of selection bias in determining which patients are suitable for hypofractionated treatments on the Extend system versus single‐fraction, frame‐based GKRS. At our center, patients selected for the Extend system tend to have good performance status and a willingness to tolerate the multiple positional measurements required during simulation and before each treatment fraction. If less favorable patients were included, it is likely the measurement uncertainty would increase, with a corresponding increase in dosimetric uncertainty to nearby OARs.

The Extend system was designed specifically to make practical hypofractionated radiosurgery treatments using the Gamma Knife. The primary rationale for treating in multiple fractions rather than single fractions is to help protect nearby organs at risk from excessive radiation damage by allowing for interfraction repair. The worst‐case increase in maximum dose to an OAR was 30.59%. In this study, the most common nearby OARs were the anterior optic pathways. For single‐fraction treatments, optic nerve tolerance has been reported to be in the range of 12 Gy to a maximum point dose.^14^, ^15^ Under the worst‐case shift observed in the study, the maximum dose to the anterior optic pathways would increase from 10.44 Gy to 15.67 Gy. However, over 3 and 5 fractions of radiation, optic nerve tolerance is estimated to be between 17 Gy and 23 Gy.^(16)^ So under the observed worst‐case shift (in the unlikely event it occurred over all treatment fractions) the optic nerves would receive a higher dose overall all fractions, but they would remain within expected tolerance. In this sense, our study helps to confirm the trade‐off between increased uncertainty with the Extend system and the ability to hypofractionate with the Gamma Knife.

Finally, the potential for positional uncertainties to cause changes in dose to target and OAR naturally leads to the question of whether treatment margins are warranted for multifraction Gamma Knife radiosurgery. Most well‐known margin formulas used in radiotherapy (the van Herk formula being one example) were developed for traditional fractionation schedules. Among their underlying assumptions is that the dose will be delivered via an infinite number of small fraction treatments.^(17)^ The systemic component of uncertainty in these situations dominates the margin calculation because over many treatments the random components of uncertainty tend to average out. In a hypofractionated treatment technique, the random component of uncertainty will play a much larger role, and may be dependent on patient‐specific and clinic‐specific variables. While several centers are working to devise population‐based margin formulas for radiosurgery settings,^(18)^ it may be that appropriate margins may need to be determined empirically on an institutional basis.

## V. CONCLUSIONS

Positional uncertainties when performing multifraction Gamma Knife radiosurgery are small (<1 mm) and are comparable to published uncertainties for frame‐based systems. As in the case of frame‐based, single‐fraction treatments, the steep dose gradients characteristic of GKRS mean that the dosimetric consequences of positional uncertainties should be considered as part of treatment planning with the Gamma Knife Extend system. However, these dose uncertainties should be evaluated in the context of tumor response and OAR tolerance for hypofractionated treatment scenarios where any increase in dose may be tempered by the increased protection hypofractionation provides to normal tissue.

## ACKNOWLEDGMENTS

David Schlesinger receives research support from Elekta Instruments, AB.

## COPYRIGHT

This work is licensed under a Creative Commons Attribution 4.0 International License.
